# Expanding Cystic Fibrosis Registries to the Rest of the World

**DOI:** 10.1002/ppul.71445

**Published:** 2026-01-08

**Authors:** Lutz Naehrlich

**Affiliations:** ^1^ Department of Paediatrics Justus‐Liebig‐University Giessen Giessen Germany

**Keywords:** cystic fibrosis, epidemiology, global health, patient registry

## Abstract

**Challenge:**

Cystic fibrosis (CF) is a global challenge. The epidemiological knowledge is incomplete and focused on patient registries in the United States, Canada, Europe, Australia and New Zealand, Brazil, and South Africa. To complete the global picture of CF, we have to learn from each individual with CF, as well as each cohort and population.

**Solution:**

Structured, harmonized, quality‐controlled, and sustainable patient registries are needed worldwide to complete this picture and give every person with CF the opportunity to be part of it.

**Requirements:**

Commitment, trust, transparency, and independence are key elements to building and running a sustainable registry. Combining the experience of existing registries with the commitment of persons with CF and caregivers around the globe could build the basis for more complete data collection. In doing so, we must strike a balance between the quantity and quality of data. The European CF Society Patient Registry Partnership Project for CF registries in low‐ and middle‐income countries outside the World Health Organization European Region is an example of bridging the gaps and allowing broader registry participation.

**Conclusion:**

“Do not call the global CF registry a dream, call it plan,” and let's start with the first steps and get involved in the global CF community.

Cystic fibrosis (CF) is a global challenge and has been reported in 96 countries worldwide [1]. Of the estimated 188,336 people with CF (pwCFs), 111,767 have been diagnosed; 91% of all diagnosed pwCFs have been reported by patient registries in the US [2], Canada [3], Brazil [4], Europe (covering 40 countries in the World Health Organization [WHO] European Region) [5], South Africa [6], Australia [7], and New Zealand [8], (Figure 1). For other countries, epidemiological knowledge is based on published literature (42 countries) and CF experts (7 countries), but no data are available for 63 countries. These numbers reflect the predominance of CF in Caucasian populations, but also the different levels of awareness and access to diagnosis and therapy around the world [9]. In recent years, improved life expectancy and a reduced disease burden have been documented by established patient registries in the US [2], Canada [3], and Europe [5] (including national registries from France [10], Germany [11], Ireland [12], Italy [13], the Netherlands [14], and United Kingdom [15]), South Africa [6], and Australia [7]. However, these findings account for only 59% of the estimated cases of CF worldwide, and more than 75,000 patients have not been diagnosed [1]. This underlines how incomplete our global picture of CF is and how much effort is needed to close these gaps.

**Figure 1:**
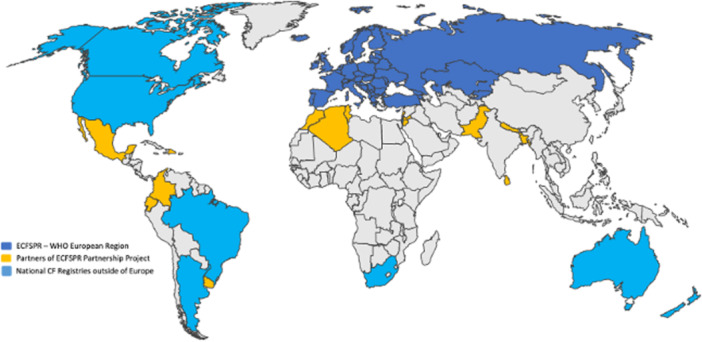
Cystic fibrosis registries around the Globe 2025. [Color figure can be viewed at wileyonlinelibrary.com]

## 1 Can CF Registries Make a Difference?

CF registries alone cannot solve the problems of care structures or access to medication [16], but they are important to achieving these goals and helping improve the quality of care [17]. The documentation of each pwCF in a CF registry is a critical review that can identify individual challenges, gaps, and the room for diagnostic and therapeutic improvements, and can be seen as part of quality control. Analysis of a cohort at the center and country level gives the opportunity to see a broader picture regarding age distribution, age at diagnosis, disease progression, and the burden of therapy, and helps to identify opportunities for improvement in the structure and diagnostic/therapeutic strategy over time. Comparisons between centers in a country or between countries offer the possibility to discuss different diagnostic/therapeutic strategies, to learn from the best, and to improve care and outcomes over time [18, 19]. The registry can be key to building up and strengthening the CF community in a country and internationally. The Twinning project of the European CF Society (ECFS) [20] and the international cooperation between US centers and other countries, such as Jordan [21], Egypt [22], and India [23], and the quality project in Turkiye [24] are examples of international data‐driven cooperation.

CF registries can also be used to inform pwCFs and their families about CF in their own center/country, to raise awareness in the local community with the help of patient advocacy groups (or to build one), to raise awareness among health authorities and industry, to improve diagnosis and therapy, and even to establish CF care. South Africa established a CF registry in 2018 [6], documenting the progress and gaps [25] and advocating in cooperation with South African CF Association for secured access to CFTR modulators [26].

From a global perspective, our epidemiological knowledge is limited mostly to high‐income countries and is incomplete with respect to global genetic diversity, access to care, burden of disease, and life expectancy. Each visible CF population helps enhance our knowledge and to build bridges to close the gaps in knowledge, diagnosis, and therapy [27]. The CFTR2 database includes 122,935 pwCFs from 55 countries and determines and assigns the disease liability of CFTR variants [28]. The CF Registry Global Collaboration led by Anne Stephenson in Canada contributed to several COVID publications on up to 6500 cases from 33 countries [29]. Both are examples of how the global community takes advantage of national and international registry collaboration.

## 2 What Are the Essential Components for Setting up a CF Registry?

Essential to setting up a CF registry is the commitment of the caregiver to spend resources (mostly time) to set up and contribute to the registry at the center level. Commitment, trust, transparency, and independence are the essential elements of registries [30]. The registry must secure the privacy rights of each pwCF and use the data to improve diagnosis and therapy in the best interests of patients. For each step of data collection, informed consent from each pwCF and/or their parent(s) is critical. Transparency, independence, and governance of the registry are the basis for obtaining the trust of pwCFs and their caregivers. This includes the self‐obligation of each registry to report the collected data in publicly accessible, up‐to‐date annual reports and the use of data for research. Each centre should be seen as a data owner with the right to use their data according to informed consent. On a national and international level, transparent governance rules on data usage are needed. This should incl. publication guidelines (incl. an internal review board and process), the obligation to inform about the research request, and the right to opt‐in/out on an international level. The registry handles highly sensitive data from the CF community, and the use of the data should be independent from any conflicts of interest, either financial or commercial.

## 3 How Can a CF Registry be Set up?

**Figure 2:**
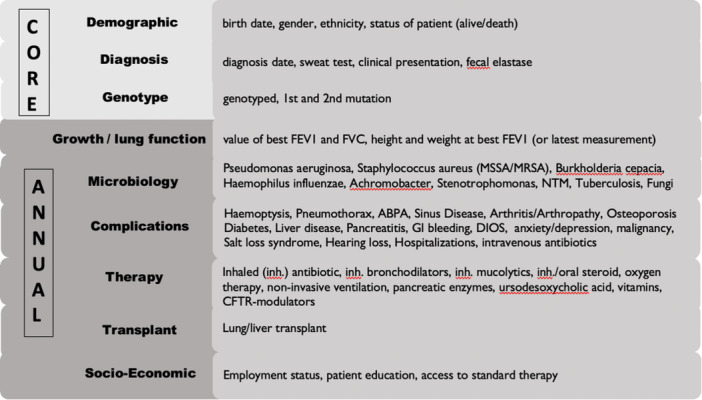
Minimal dataset according CFF/ECFSPR proposal [33]. [Color figure can be viewed at wileyonlinelibrary.com]

A CF registry should be set up as an organized system to collect and disseminate high‐quality data on patients with a confirmed diagnosis of CF using a harmonized set of variables, definitions, and references. Harmonization is critical for comparing results. There is a broad consensus about the core data (demographic, diagnostic, genotype) and the most important annual follow‐up data (growth/lung function, microbiology, complications, therapy) for CF, but even among existing registries, there is divergence that complicates data sharing and comparisons [25, 31, 32]. Consequently, in 2021, the CF Foundation (CFF) and ECFS Patient Registry (ECFSPR) worked on a so‐called “minimal dataset” [33] to set up harmonized data collection for new registries. This annual dataset reflects the balance between the quality and quantity of data and considers the limited resources for data collection (Figure 2). Encounter‐based data collection combined with high data completeness, quality, and reporting tools can provide better individual and cohort overviews (examples CFF‐Registry, Germany), but is rarely implemented due to the significantly higher efforts involved. Both centralized (web‐based generic tools, such as REDCap or Castor, or registry‐specific tools, such as ECFSTracker) and decentralized (Excel templates or REDCap) data collection tools have been used; web‐based data collection combined with centralized data storage and management allows centralized pseudonymization for each pwCF to avoid duplicates and centralized data quality control, but comes with a higher need for resources. Uploading decentralized data collections to a centralized data collection tool, including data quality checks, is offered, for example, by the ECFSPR. Data management is critical for improving the completeness and quality of data, securing continuous work, taking responsibility for the use of data, and disseminating results [30]. To minimize data errors, registries should incorporate built‐in validation rules, range checks, and automated consistency checks across forms, along with audit trails to track data entry and corrections. Regular feedback reports to participating centers may further support data verification and continuous quality improvement, as data quality is critical for each use case.

## 4 How Can Resources for a CF Registry be Raised?

It takes a village to set up and run a CF registry, and resources are a critical barrier. The multiple use cases of registries are a blueprint for the collaboration of caregivers with patient advocacy groups, health authorities, or independent associations to raise the necessary resources. Grants from industry must be unrestricted to secure privacy rights and independent analysis. For countries in the WHO European Region, the ECFSPR offers a free data collection framework, including uploading to national registries, data management, and annual reporting. This approach has led to 26 countries without a national registry being added to the 17 existing national registries and to completing the picture of CF in the WHO European Region [5].

## 5 The ECFSPR Partnership Project for Registries Outside the WHO European Region

The ECFS decided to issue a call in June 2025 for a Partnership Project for low‐ and middle‐income countries outside the WHO European Region [34]. The 5‐year project (2025–2030) will cover training and access to web‐based data collection software, centralized data storage, data management, quality control, and annual public reporting. The governance of the ECFSPR sets the framework, and the centers have to take care of ethical votes, data sharing agreements, informed consent, data entry, and quality control. Fifteen countries have been selected as partners and will contribute after successful ethical approval to the worldwide epidemiology knowledge in the near future (Sep. 2025) (Figure 1).

## 6 Conclusion

CF registries have a major impact on epidemiological knowledge. We learn from each pwCF, cohort, and population, as well as the global picture of CF. To complete this picture and give every pwCF the opportunity to be a part of it, structured, harmonized, quality‐controlled, and sustainable patient registries are crucial. Commitment, trust, transparency, and independence are key to building and running a sustainable registry. Combining the experience of existing registries with the commitment of pwCFs and their caregivers around the world could lead to more complete data collection. In doing so, we must strike a balance between the quantity and quality of data. The ECFSPR Partnership Project for CF registries in low‐ and middle‐income countries outside the WHO European Region is an example of bridging gaps and allowing broader registry participation. “Do not call the global CF registry a dream, call it a plan,” and let's start with the first steps and get involved in the global CF community.

## Author Contributions

**Lutz Naehrlich:** conceptualization, writing – original draft.

## Funding

The author received no specific funding for this work.

## Conflicts of Interest

L.N. receives institutional funding from the European Cystic Fibrosis Society for Pharmacovigilance studies and from Vertex Pharmaceuticals for clinical study participation.

## Data Availability

The author has nothing to report.
